# Acute Peritonitis: A Mimic of Malignancy-Related Ascites in a Patient With Peritoneal Carcinomatosis

**DOI:** 10.7759/cureus.60705

**Published:** 2024-05-20

**Authors:** Henry Egbuchiem, Onoja-Frederick Okwori, Joseph Amoah, Sedina Asafu-Adjaye, Mohammed Mazumder

**Affiliations:** 1 Internal Medicine, University Hospitals, Geauga Medical Center, Cleveland, USA; 2 Internal Medicine, University Hospitals, Cleveland Medical Center, Cleveland, USA; 3 Gastroenterology, University Hospitals, Geauga Medical Center, Cleveland, USA

**Keywords:** malignant ascites, ascites, peritonitis treatment, bacterial peritonitis, peritonitis

## Abstract

Peritonitis, an inflammation of the peritoneal cavity, can be caused by various factors. The presence of ascites in a cancer patient is concerning for either metastasis or advanced cancer. Diagnosing acute peritonitis in a patient with cancer-related ascites can be quite challenging and often requires additional diagnostic procedures, such as paracentesis, to confirm the diagnosis and identify the exact cause of the ascites. Even with paracentesis, determining the exact cause of ascites can be a diagnostic challenge. Peritoneal carcinomatosis, with a poor survival rate, can originate from the peritoneal lining itself or result from intra-abdominal cancer, and trying to determine its origin can be difficult. We present the case of a 68-year-old female patient with a known history of cancer experiencing worsening ascites and peritonitis.

## Introduction

Peritonitis is an inflammation of the inner lining of the abdominal cavity, also known as the peritoneum. Malignancy-related ascites (MRA) are responsible for only about 7% of ascites in the United States compared to 85% in patients with cirrhosis and portal hypertension [1]. It is not uncommon to find MRA in individuals with the following solid tumors: breast, lung, liver, pancreas, ovaries, and colon malignancies [2]. Malignancy-related ascites, a discreet disease entity, is often confused with peritoneal carcinomatosis. It is essential to differentiate between MRA and peritoneal carcinomatosis, as the former results from intra-abdominal malignancy leading to ascites. At the same time, the latter is a distinct form of peritoneal cancer originating from the peritoneum. Both conditions can present with nonspecific symptoms such as ascites, abdominal discomfort, and signs of constipation, therefore making it challenging to determine the exact cause of ascites in patients with malignancy.

As per Runyon et al. [3], liver cirrhosis is the leading cause of ascites, responsible for 81% of all cases. Cancer follows at approximately 10% and heart failure at around 3%. Tuberculosis accounts for 2%, while dialysis and pancreatic disease each contribute 1% to ascites cases. Additionally, they hypothesized that only about two-thirds of patients with malignancy-related ascites have peritoneal carcinomatosis.

The overlap between ascites and peritoneal carcinomatosis can complicate the diagnostic process. Some authors have described peritoneal carcinomatosis as a late manifestation of gastrointestinal cancers, which may initially be asymptomatic but later lead to nonspecific symptoms such as abdominal pain, nausea, bloating, and weight loss as the disease progresses [4]. Research by Ross et al. [5] revealed that secondary peritonitis accounts for 1% of all global hospital admissions and is a cause of sepsis in the ICU, with an overall mortality rate of 6%. Therefore, prompt diagnosis with paracentesis and early initiation of antimicrobials is crucial. This case study presents a challenge in determining the exact cause of ascites, as a patient with peritoneal carcinomatosis and malignancy-related ascites developed peritonitis following an abdominal procedure.

## Case presentation

The patient is a 68-year-old female with a medical history significant for papillary renal cell cancer (type 1, grade 2 tumor with spread to the omentum) status post right nephrectomy and chemotherapy (cabozantinib 40 mg, Lenvima 8 mg, and nivolumab) with a tunneled indwelling peritoneal catheter placement (inserted one year prior to presentation), chronic obstructive pulmonary disease on 2 L nasal cannula home oxygen at night, chemotherapy-induced cardiomyopathy (cabozantinib), and heart failure with preserved ejection fraction who presented to the hospital on account of one-week history of lower quadrant abdominal pain and nausea. She reported a temporal history of commencement of abdominal pain after the tunneled indwelling peritoneal catheter was replaced one week earlier, with an associated history of 30 mL fluid drainage from the catheter. She describes abdominal pain as 10/10 intensity sharp, diffusely around the lower abdomen, more on the right lower abdominal quadrant. She states that the pain was only mildly reduced by using opioids (oxycodone 10 mg, orally, every four hours). There is an associated history of nausea, decreased appetite, and an unintentional weight loss of 22 kg in the last year. Past surgical history is significant for right nephrectomy and tubal ligation. She is allergic to trimethoprim/sulfamethoxazole and is a former smoker (she has a 20-pack-year smoking history and stopped smoking three years ago). Vital signs showed a temperature of 36.2°C, heart rate of 87 beats/minute, respiratory rate of 18 cycles/minute, blood pressure of 153/75 mmHg, and oxygen saturation (SpO2) of 92% on room air. Physical examination is significant for a distended abdomen and a draining catheter in the right lower abdominal quadrant, draining thick, yellowish discharge. The surrounding skin is clean, without any erythema, purulent discharge, or other visible skin lesions. There were normoactive bowel sounds on auscultation with tenderness to palpation of lower abdominal quadrants, positive rebound tenderness without guarding, and a negative Murphy's sign on palpation. The cardiopulmonary examination is unremarkable.

Laboratory data showed a blood glucose of 109 g/dL, a complete blood count with mild leukocytosis, a white blood cell (WBC) count of 12.2 (normal range: 4-11 × 10^9^/L), neutrophils of 10,660/µL (normal range: 1,500-8,000/µL), and microcytic anemia with hemoglobin of 10 g/dL (normal range: 12-16 g/dL), and mean corpuscular volume of 70 fL (normal range: 80-100 fL). The liver function test was significant for aspartate aminotransferase (AST) of 28 u/L (normal range: 9-39 u/L), alanine aminotransferase of 49 u/L (normal range: 7-45 u/L), alkaline phosphatase of 128 u/L (normal range: 33-136 u/L), total bilirubin of 0.5 ng/dL (normal range: 0-1.2 ng/dL), and serum albumin of 3.3 g/dL (normal range: 3.4-5.0 g/dL). Prothrombin time was 13.6 seconds (normal range: 9.8-12.8 seconds), and the international normalized ratio (INR) was 1.2 (normal range: 0.9-1.1). Total protein was 5.9 g/dL (normal range: 6.4-8.2 g/dL).

Paracentesis fluid analysis showed a WBC of 24,040 cells/uL (normal range: <500 cells/uL), neutrophils of 77, and polymorphonuclear (PMN) cells of >250/uL (normal range: <250/uL), with glucose of 95 mg/dL (normal range: 70-100 mg/dL), with a cloudy, yellow paracentesis fluid. Gram stain was positive for variable negative and positive organisms, and the final culture of the paracentesis fluid grew *Gordonia* species susceptible to ciprofloxacin after three days of incubation.

Pathology reports of the left pelvic mass biopsy showed neoplasm, consistent with renal cell carcinoma, papillary type, with neoplastic cells displaying bland cytologic features. Neoplastic cells are diffusely immunoreactive for paired box 8 (*PAX8*) gene, cytokeratin 7, HNF1 (homeobox), and alpha-methyl acyl-CoA racemase (AMACR), and they display patchy immunoreactivity for carbonic anhydrase IX. Neoplastic cells are negative for Wilms tumor (WT)-1, estrogen receptor, and progesterone receptor. The neoplastic cells also show wild-type p53 (tumor protein) immunostaining consistent with a type 1 papillary renal carcinoma.

**Figure 1:**
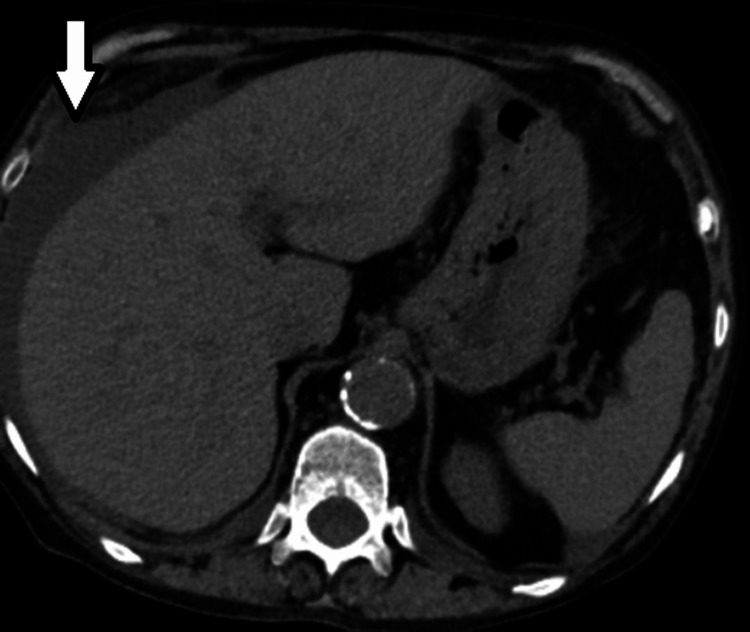
Perihepatic ascites (white arrow)

**Figure 2:**
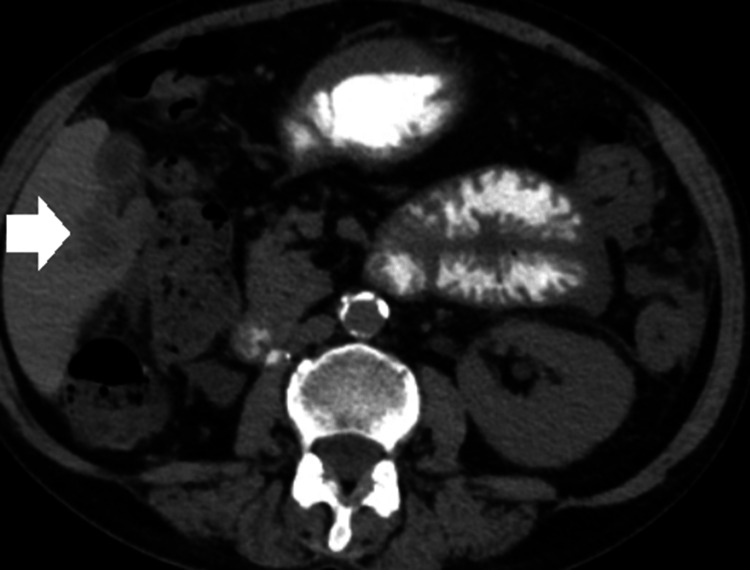
Liver metastasis (white arrow)

**Figure 3:**
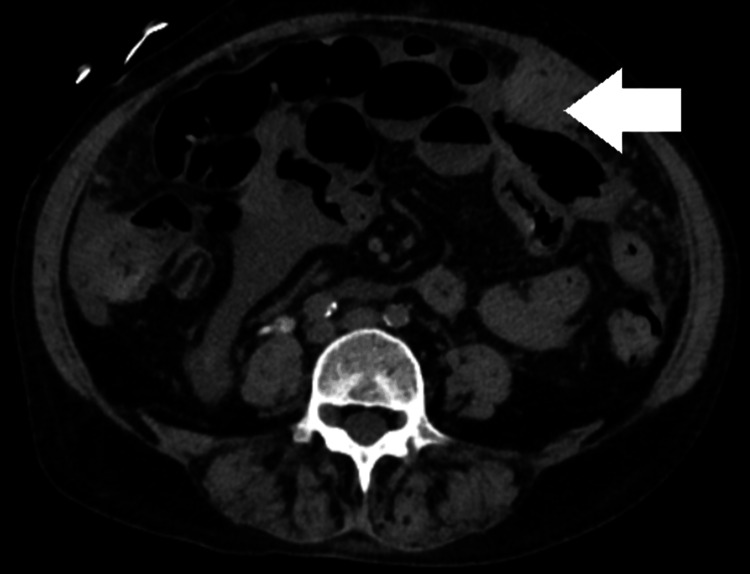
Peritoneal carcinomatosis measuring 32 × 22 mm (white arrow)

**Figure 4:**
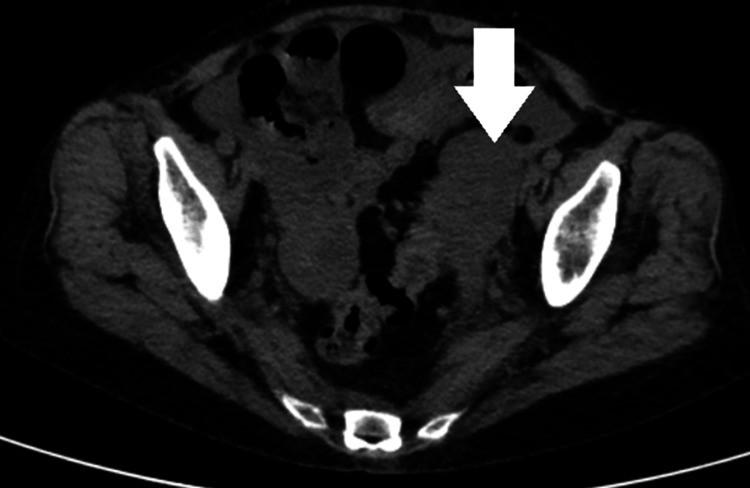
Left-sided retroperitoneal tumor (white arrow)

Figure 1 shows perihepatic ascites. Figure 2 shows metastatic liver lesions. Figure 3 shows peritoneal carcinomatosis and interval increase in ascites. Figure 4 shows left-sided retroperitoneal mass.

A diagnosis of acute secondary peritonitis was made, and the patient was subsequently treated with IV antibiotics (ciprofloxacin 500 mg twice daily); her abdominal pain was controlled with her home regimen (oxycodone 10 mg every four hours), for which she improved and was discharged two days after admission. At her follow-up clinic visit one week after discharge, she reported a complete resolution of her symptoms.

## Discussion

Peritonitis, peritoneal carcinomatosis, liver pathologies, heart failure, and malignancy all result in the formation of ascites. Paracentesis with serum-ascites fluid analysis helps evaluate and ultimately determine the etiology of ascites in patients with increased abdominal discomfort, tenderness, distention, swelling, pyrexia, and altered mental status. Calculating serum-ascites albumin gradient (SAAG) is fundamental in classifying the etiology of ascites. High albumin gradient (SAAG greater than or equal to 1.1 g/dL) is obtainable in the following conditions: heart failure, constrictive pericarditis, alcoholic hepatitis, cirrhosis, Budd-Chiari syndrome, portal vein thrombosis, idiopathic portal fibrosis, and massive hepatic metastasis. In comparison, common causes of a low albumin gradient (SAAG less than 1.1 g/dL) include nephrotic syndrome, serositis, pancreatitis, peritoneal tuberculosis, and peritoneal carcinomatosis.

**Figure 5:**
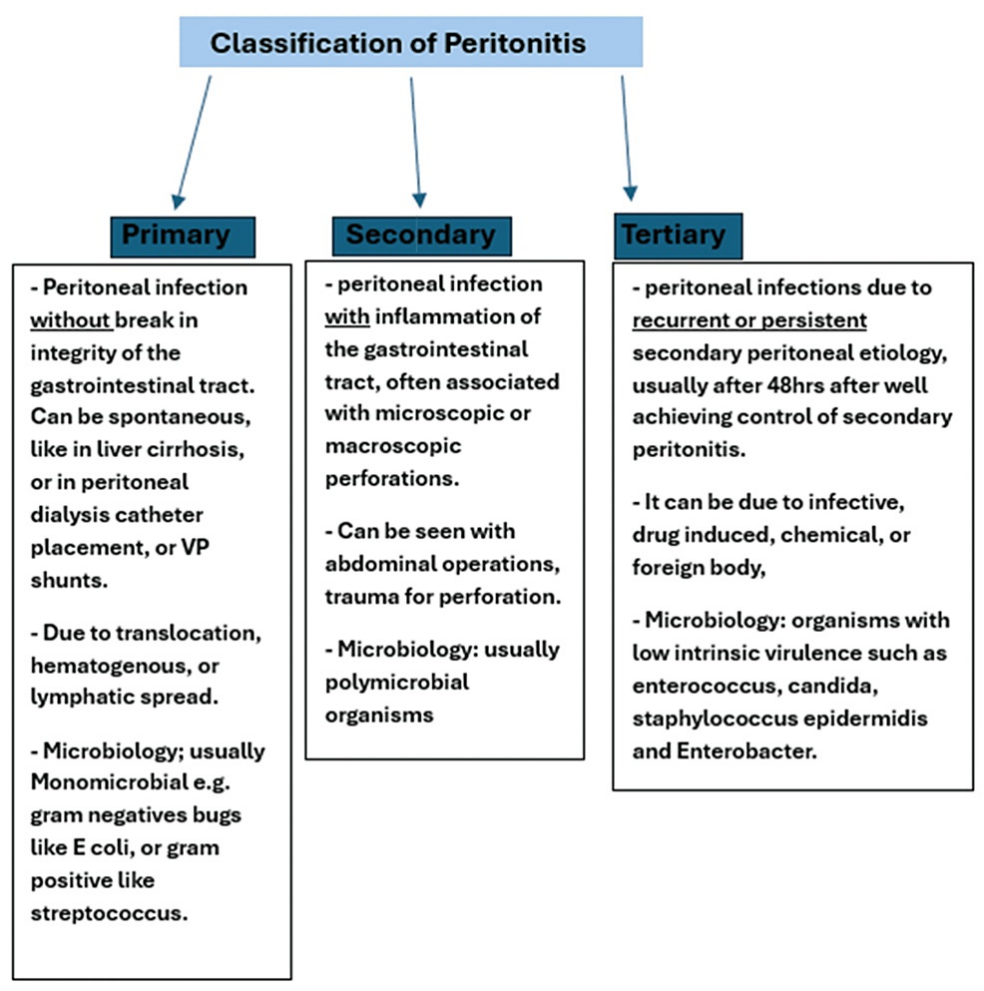
Classification of peritonitis into primary, secondary, and tertiary VP: ventriculoperitoneal

Primary peritoneal infection can occur without a gastrointestinal tract defect after direct bacterial translocation, hematogenous spread, or iatrogenic abdominal cavity contamination [6]. Although the advent of antibiotics has reduced the occurrence of peritonitis, after a recent abdominal procedure, the suspicion of bacterial translocation and subsequent infection of the peritoneum is not uncommon [6]. Primary peritoneal infection is different from spontaneous bacterial peritonitis (SBP), a life-threatening complication found in patients with liver disease or cirrhosis. Figure 5 shows the classification of peritonitis.

**Figure 6:**
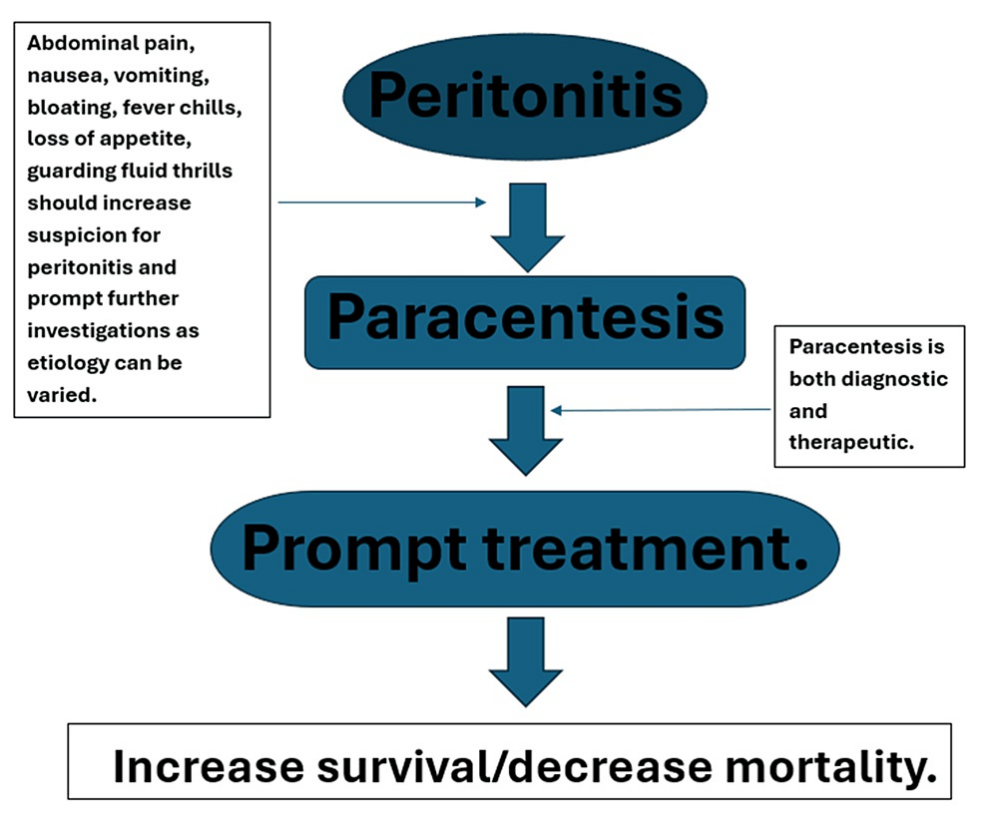
Assessment of peritonitis The illustration shows that prompt diagnosis of peritonitis leads to increased survival.

However, procedures involving the insertion of a needle or foreign object through the abdominal wall to access the peritoneal cavity, such as paracentesis and placement of abdominal drainage catheters, have their advantages (they are both diagnostic and therapeutic). Figure 6 illustrates the diagnostic framework for evaluating peritonitis. Research by Orman et al. [7] indicates that while patients undergoing paracentesis are at an increased risk of intra-abdominal bacterial infection, the procedure improves short-term survival rates. Their study, which included 17,711 eligible admissions, found that approximately 61% of their study population underwent paracentesis, ultimately resulting in a 24% reduction in in-hospital mortality (6.5% versus 8.5%; adjusted odds ratio: 0.55; 95% confidence interval: 0.41-0.74).

Although our patient discussed in the case above has a baseline collection of intra-abdominal fluid from peritoneal carcinomatosis versus malignancy-related ascites, the presence of new-onset abdominal pain, increased abdominal girth, leukocytosis on complete blood counts, increase in polymorphonuclear cells in ascites fluid of more than 250/uL, and increase in white blood cells in ascites fluid with amounts more significant than 20,000, in addition to worsening ascites fluid collection after a recent change in the abdominal drainage catheter, are indicative of infective ascites fluid, which was subsequently responsive to antibacterial therapy.

## Conclusions

Identifying the exact cause of peritonitis in patients with known or prior ascites is crucial since several peritoneal pathologies can co-exist, and the treatment of ascites varies depending on the different causes. The complexity of this case report portrays the challenges healthcare providers face as they promptly diagnose and manage patients with ascites effectively. This article discusses different types of peritonitis, highlights the diagnostic challenges, and stresses the importance of prompt paracentesis in diagnosing the cause of ascites in patients with a history of cancer. Furthermore, publishing this case report is valuable to the current medical literature as it may also encourage further research into the complex phenomenon of determining the cause of peritonitis, potentially leading to the development of new treatment approaches.
